# Elucidation of the molecular mechanism of type 2 diabetes mellitus affecting the progression of nonalcoholic steatohepatitis using bioinformatics and network pharmacology: A review

**DOI:** 10.1097/MD.0000000000039731

**Published:** 2024-09-13

**Authors:** Bo Wu, Xiaohong Lan, Ming Gao, Wei Wei, Yuekun Wang, Yang Yang, Zhiyang Yu, Min Huang, Qinyan Wu

**Affiliations:** a Department of Pharmacy, Jinling Hospital, Affiliated Hospital of Medical School, Nanjing University, Nanjing, China; b The fourth was assigned to the outpatient department, Jinling Hospital, Affiliated Hospital of Medical School, Nanjing University, Nanjing, China.

**Keywords:** bioinformatics, mechanism, network pharmacology, nonalcoholic steatohepatitis, type 2 diabetes mellitus

## Abstract

Increasing evidence suggests that patients with diabetes are at increased risk of developing nonalcoholic steatohepatitis (NASH), but the underlying mechanisms that affect the progression of NASH remain unclear. In this study, we used bioinformatics and network pharmacology methods to explore the differentially expressed genes of NASH and the related genes of type 2 diabetes mellitus, and a total of 46 common targets were obtained. Gene ontology showed that the common targets were mainly involved in biological processes such as glucocorticoid, hormone, and bacterium responses. The Kyoto Encyclopedia of Genes and Genomes enrichment analysis signal pathways were mainly in colorectal cancer, amphetamine addition, the peroxisome proliferator-activated receptor signaling pathway, and the toll-like receptor signaling pathway. The protein–protein interaction network identified 8 hub genes, and the co-expression network was analyzed to obtain 7 related functions and mutual proportions of hub genes. A total of 120 transcription factors were predicted for hub genes. Hub genes were closely related to immune cells, including neutropils and eosinophils. In addition, we identified 15 potential candidate drugs based on hub genes that are promising for the treatment of NASH. Type 2 diabetes mellitus can affect the progression of NASH by changing hormone levels and inflammatory responses through multiple targets and signaling pathways. Eight hub genes are expected to be potential targets for subsequent treatment.

## 1. Introduction

**Figure 1.**
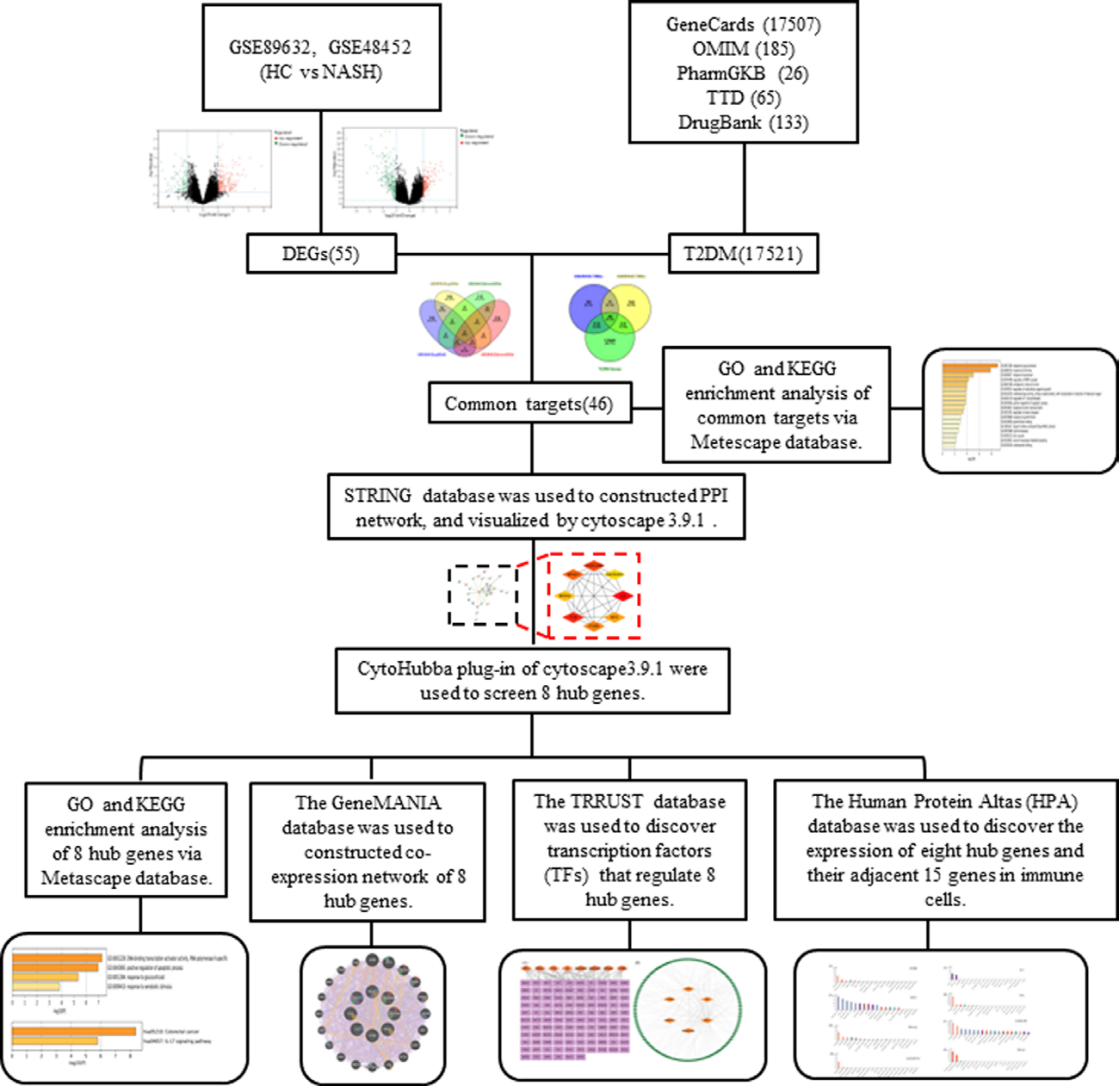
Step diagram of the research route. DEGs = differentially expressed genes, GO = Gene ontology, HC = liver control, KEGG = Kyoto Encyclopedia of Genes and Genomes, NASH = nonalcoholic steatohepatitis, OMIM = Online Mendelian Inheritance in Man, T2DM = type 2 diabetes mellitus, TDD = Therapeutic Target Database.

Nonalcoholic fatty liver disease (NAFLD) includes nonalcoholic hepatic steatosis, nonalcoholic steatohepatitis (NASH), liver cirrhosis, and hepatocellular carcinoma. NASH is formed on the basis of nonalcoholic hepatic steatosis, in which serum biochemical enzymes exceed the upper limit of normal, or liver biopsy histopathology shows hepatocyte steatosis >5%, accompanied by inflammation and hepatocyte injury (such as ballooning), and other causes of hepatic steatosis are excluded.^[1]^ NASH is an important link in the development of liver fibrosis (LF), liver cirrhosis, and even hepatocellular carcinoma. Relevant studies have shown that NASH accounts for 41.4% to 54.0% of NAFLD patients. NAFLD patients with obesity, hyperlipidemia, and type 2 diabetes mellitus (T2DM) usually have severe liver histological damage, and the detection rate of NASH and progressive LF is high.^[2]^ NASH has become the second most common liver disease in the field of liver disease in the 21st century. It is expected that NASH may gradually become one of the main pathogenic factors for end-stage liver disease, liver transplantation, and primary liver cancer in the next 10 years.^[3]^ As mentioned above, the risk and severity of NASH in patients with T2DM are much higher than those in normal people. However, how T2DM affects the progression of NASH and its regulatory mechanisms are still unclear, and there is still a lack of targeted therapeutic drugs. In recent years, bioinformatics and network pharmacology have been widely used to elucidate “disease-disease,” “drug-disease,” “gene-disease,” and “protein-disease” interactions.^[4–6]^ Therefore, this study aims to explore the relationship between T2DM and NASH through bioinformatics and network pharmacology analysis, explore its potential regulatory mechanism, and provide a certain theoretical basis and innovative perspective for the joint prevention and treatment of T2DM and NASH and the research of new drugs. The study flow is shown in Figure 1.

## 2. Materials and methods

### 2.1. Selection of transcriptome microarray datasets

The Gene Expression Omnibus database contains a large number of high-throughput sequencing datasets. NASH was searched in the Gene Expression Omnibus database, and the NASH-related transcriptome datasets GSE48452 and GSE89632 were selected. Data collection, diversity expression gene screening, functional enrichment analysis, protein–protein interaction (PPI) network construction, and core target gene screening date of the solstice are January 12, 2024. The GSE48452 dataset included 14 NASH patients (4 males and 10 females; age ranged from 30 to 58 years, with an average age of 45.4 years) and 12 healthy controls (5 males and 7 females; age ranged from 28 to 80 years, with an average age of 44.8 years). The GSE89632 dataset included 19 NASH patients (9 males and 10 females; age ranged from 23 to 68 years, with an average age of 43.5 years) and 24 healthy controls (11 males and 13 females; age ranged from 23 to 58 years, with an average age of 37.2 years).^[7]^

### 2.2. Analysis of differentially expressed genes and acquisition of common targets

The GE2R online analysis tool was used to analyze the differentially expressed genes (DEGs) in the 2 datasets GSE48452 and GSE89632, and the screening parameters were set as |logFC|≥1 and adjusted *P* < .01. The targets associated with T2DM were retrieved from the GeneCards (https://www.genecards.org/), Online Mendelian Inheritance in Man (https://omim.org/), PharmGKB (https://www.ph-armgkb.org/), Therapeutic Target Database (https://db.idrblab.net/ttd/), and DrugBank (https://go.drug-bank.com/) databases using the keyword “Type 2 Diabetes Mellitus.” Subsequently, the intersection of DEGs and T2DM-related genes was obtained by using the Venny2.1 tool (https://bioinfogp.cnb.csic.es/to-ols/venny/index.html) to obtain the common targets of T2DM and NASH.^[7–13]^

### 2.3. PPI network construction and hub gene screening

The PPI network was constructed using the protein interaction network analysis platform STRING (https://cn.string-db.org/),^[14,15]^ and the PPI network model was screened by the CytoHubba plug-in in Cytoscape 3.5.0.^[16]^ The maximal clique centrality algorithm was used to visualize the top 8 hub genes.

### 2.4. Gene Ontology function and Kyoto Encyclopedia of Genes and Genomes pathway enrichment analysis

Target genes were uploaded to the Metascape database (a gene annotation analysis platform) to obtain the Gene Ontology (GO) and Kyoto Encyclopedia of Genes and Genomes (KEGG) enrichment analyses of the common targets and Hub genes.^[17,18]^

### 2.5. Construction of the hub gene co-expression network and its expression in immune cells

To understand the correlation between hub genes, we used GeneMANIA (http://genemania.org/) to construct the hub gene co-expression network. GeneMANIA is an online analysis database that can predict the function of target genes and gene sets.^[19,20]^ The Human Protein Atlas (HPA) database was used to mine the expression differences of hub genes in various immune cells. HPA (https://www.proteinatlas.org/) integrates various omics technologies to map human proteins, involving various cells, tissues, and organs of the human body. The resources in the database are open access, allowing researchers to freely access data to explore the mysteries of life.

### 2.6. Prediction of transcription factors associated with hub genes

To find out which transcription factors (TFs) regulate the hub genes, we used the TRRUST database (https://www.grnpedia.org/trrust/) to predict the TFs of hub genes and constructed the hub genes-TFs regulatory network. The data source for the TRRUST database is published articles, which document experimental studies on transcriptional regulation to identify common TF target genes and regulatory relationships between TFs.^[21]^

### 2.7. Prediction of candidate drugs associated with hub genes

The drug–gene interaction database (https://dgidb.org/) collects information on drug–gene interactions and pharmacogenomes, making it easy for users to browse, search, and filter. We entered gene names, searched for drug–gene interactions, mined small molecule compounds with close links to hub genes, and then constructed a compact-hub gene network.^[22]^

### 2.8. Ethical review

In February 2023, the National Science and Technology Ethics Committee and The State Council issued the Ethical Review Measures for Life Science and Medical Research involving Human Beings. It was mentioned in the Act that “research using public databases can be exempted from ethical review.” The data of this study were from public databases and did not involve human and animal experiments, so there was no need to provide an ethical approval form.

## 3. Results

### 3.1. Screening results of common genes

**Figure 2.**
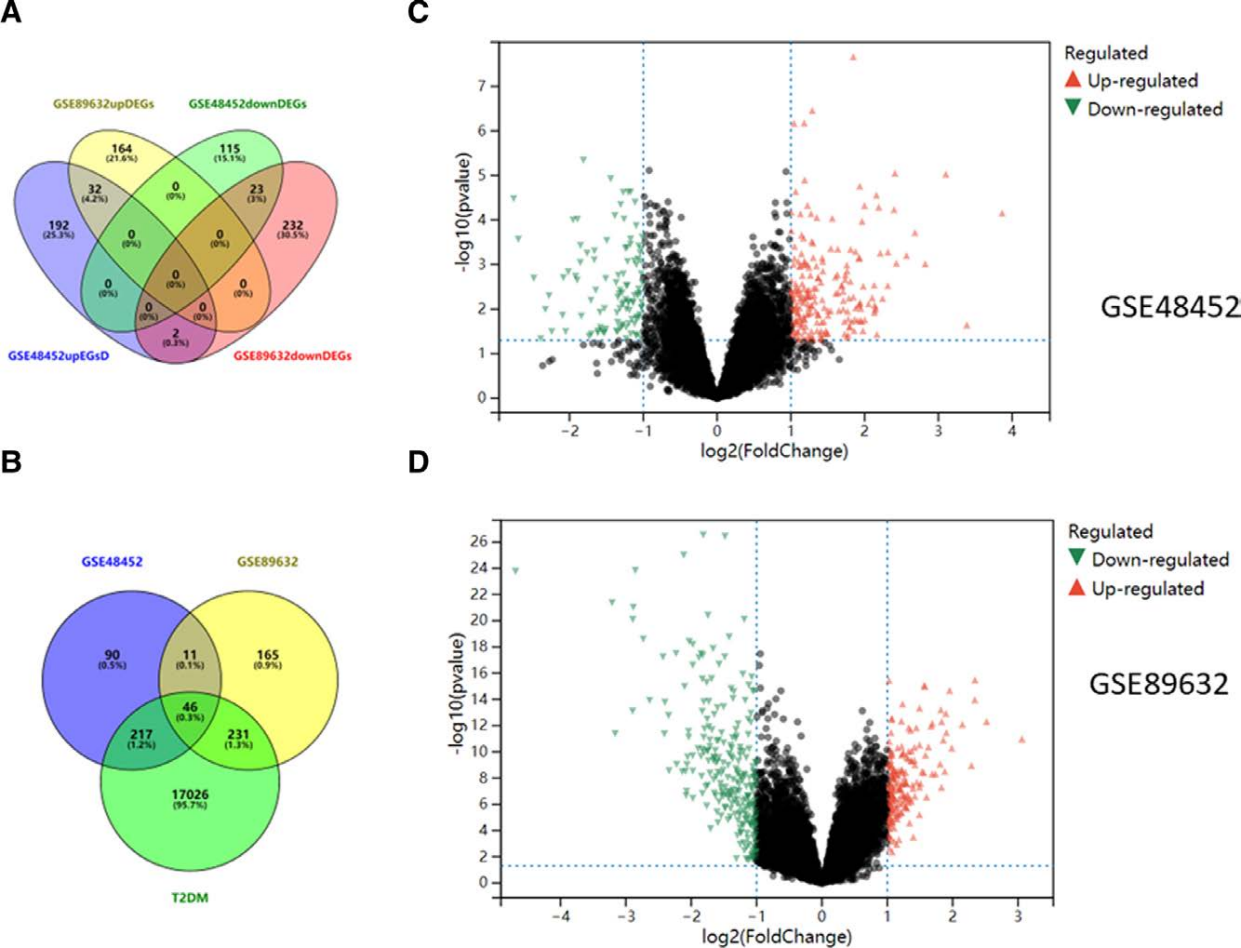
Acquisition of common genes. (A) Analysis of DEGs in NASH; (B) intersection genes of T2DM and NASH; (C) volcanic map analysis of GSE48452; (D) volcanic map analysis of GSE89632. DEG = differentially expressed gene, NASH = nonalcoholic steatohepatitis, T2DM = type 2 diabetes mellitus.

Three hundred sixty-four DEGs of NASH were obtained from the GSE48452 dataset, including 247 upregulated genes and 146 downregulated genes. Four hundred fifty-three DEGs of NASH were obtained from the GSE89632 dataset, including 196 upregulated genes and 257 downregulated genes. Finally, the intersection of the upregulated genes and downregulated genes in the 2 datasets was obtained to obtain 32 upregulated genes and 23 downregulated genes (Fig. 2A). Gene expression from both datasets is presented as a volcano plot (Fig. 2C, D). Seventeen thousand five hundred twenty-one targets related to T2DM were retrieved from the GeneCards, Online Mendelian Inheritance in Man, PharmGKB, Therapeutic Target Database, and DrugBank databases and intersected with the above DEGs to obtain 46 common targets, indicating that these targets may be involved in regulating the development of NASH (Fig. 2B).

### 3.2. Construction of a common gene expression network and screening of core target genes

**Figure 3.**
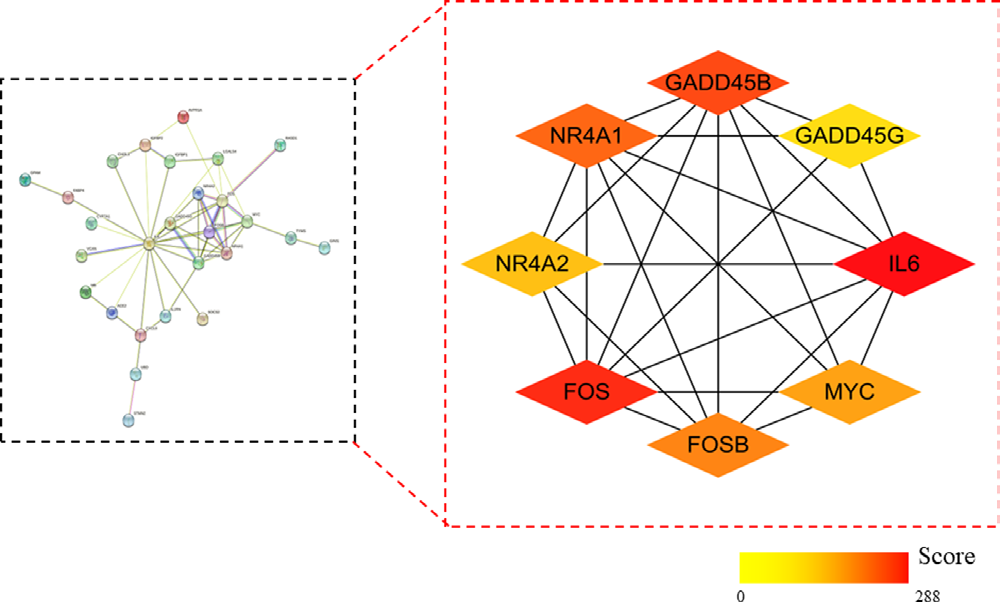
Construction of PPI networks and screening of hub genes. FOS = Fos Proto-Oncogene, AP-1 Transcription Factor Subunit, FOSB = FosB Proto-Oncogene, AP-1 Transcription Factor Subunit, GADD45B = Growth Arrest and DNA Damage Inducible Beta, GADD45G = Growth Arrest and DNA Damage Inducible Gamma, IL6 = interleukin 6, MYC = MYC Proto-Oncogene, NR4A1= Nuclear Receptor Subfamily 4 Group A Member 1, NR4A2 = Nuclear Receptor Subfamily 4 Group A Member 2.

The 46 common target genes were input into the STRING platform to construct the PPI network and then imported into Cytoscape 3.9.1 for adjustment and arrangement. A total of 27 protein interaction network nodes and 53 edges were found. The maximal clique centrality algorithm in the CytoHubba plug-in was used to screen out the top 8 hub genes as follows: interleukin 6 (IL6), Fos Proto-Oncogene, AP-1 Transcription Factor Subunit (FOS), Growth Arrest and DNA Damage Inducible Beta (GADD45B), Nuclear Receptor Subfamily 4 Group A Member 1 (NR4A1), FosB Proto-Oncogene, AP-1 Transcription Factor Subunit (FOSB), MYC Proto-Oncogene (MYC), Nuclear Receptor Subfamily 4 Group A Member 2 (NR4A2), and Growth Arrest and DNA Damage Inducible Gamma (GADD45G), and the results were visualized (Fig. 3).

### 3.3. GO and KEGG enrichment analysis of common genes

**Figure 4.**
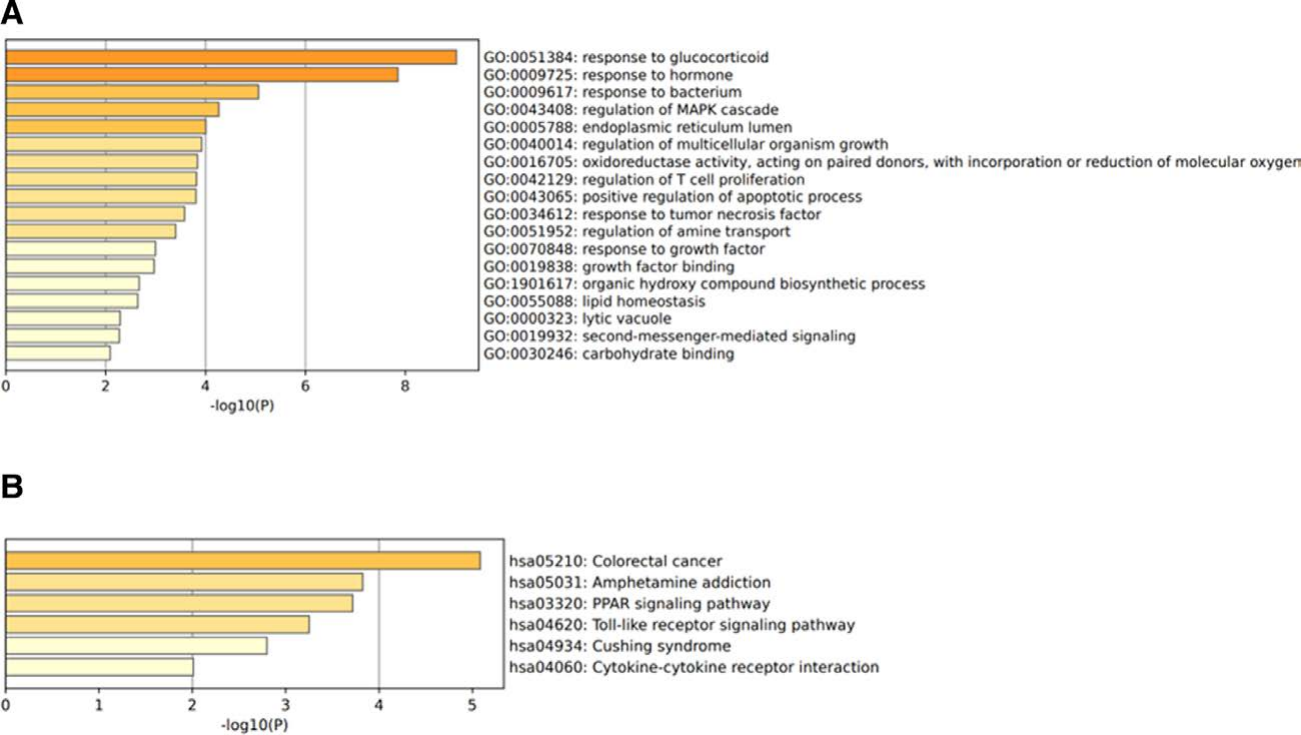
GO and KEGG enrichment analysis of common genes was performed using Metascape. (A) Terms for the GO of common genes; (B) terms for the KEGG of common genes. GO = Gene ontology, KEGG = Kyoto Encyclopedia of Genes and Genomes, MAPK = mitogen-activated protein kinase.

GO and KEGG enrichment analyses of 46 common genes were performed by the Metascape database. GO functions were mainly focused on response to glucocorticoid (GC), response to hormone, response to bacterium, regulation of mitogen-activated protein kinase cascade, endoplasmic reticulum lumen, and regulation of several aspects of multicellular organism growth. KEGG metabolic pathway analysis showed that common genes were mainly enriched in colorectal cancer, amphetamine addition, the peroxisome proliferator-activated receptor (PPAR) signaling pathway, and the toll-like receptor signaling pathway (Fig. 4).

### 3.4. Enrichment analysis of hub genes and their co-expressed gene networks

**Figure 5.**
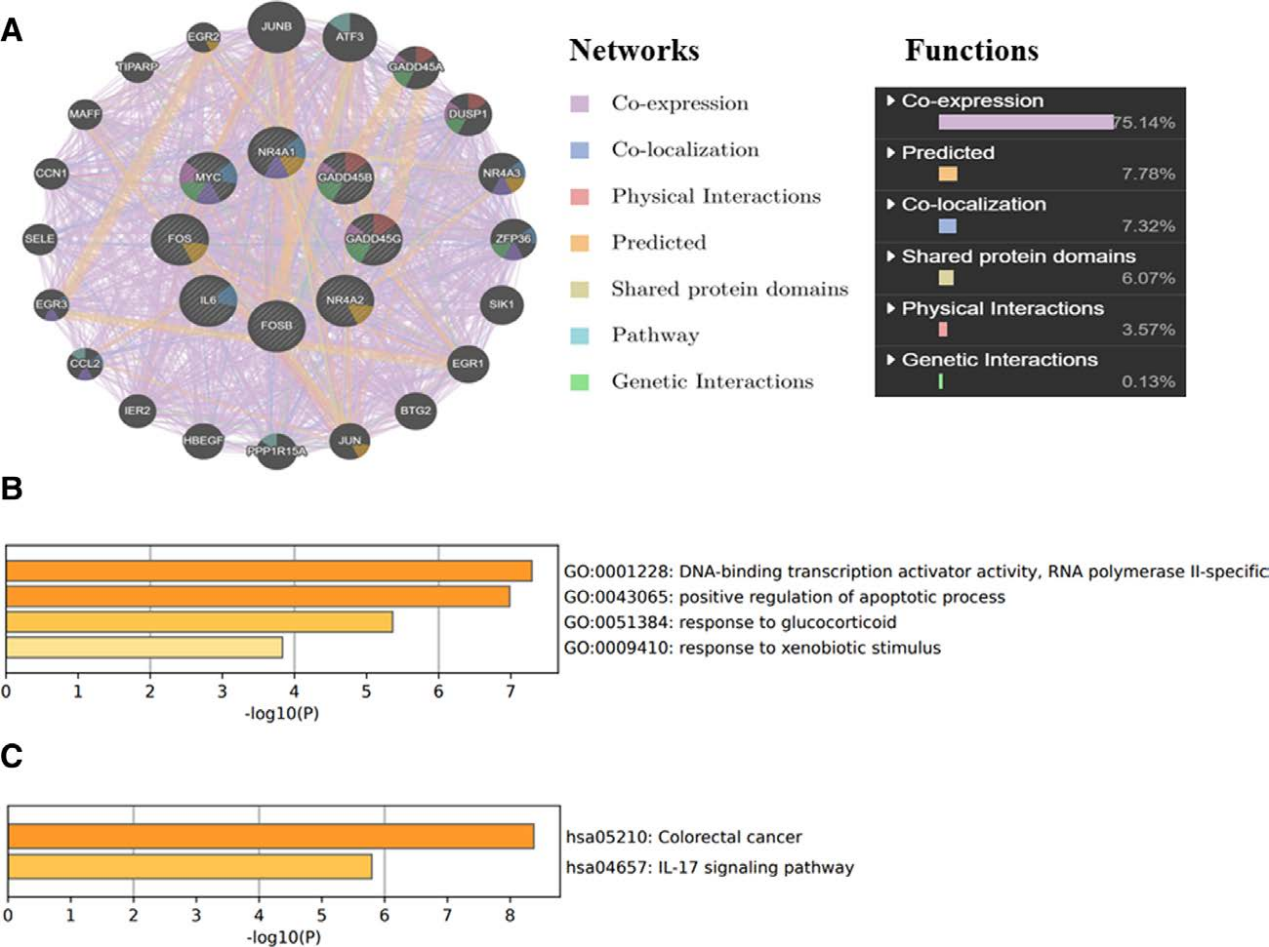
Analysis of hub genes and their adjacent associated genes. (A) Functional analysis of the PPI network and its 8 hub genes; (B) terms for the GO of hub genes; (C) terms for the KEGG of hub genes. GO = Gene ontology, IL = interleukin, KEGG = Kyoto Encyclopedia of Genes and Genomes.

GeneMANIA software was used to analyze the co-expression network of 8 hub genes, and the 7 related functions and mutual proportions of hub genes were obtained, including the co-expression of 75.14%, the prediction relationship of 7.78%, the co-localization of 7.32%, the shared protein domains of 6.07%, the physical interactions of 3.57%, and the genetic interactions of 0.13% (Fig. 5A). The GO functions of the 8 hub genes were mainly concentrated in positive regulation of the apoptotic process, response to GCs, and response to xenobiotic stimuli. Moreover, the signaling pathways were mainly concentrated in colorectal cancer and the IL-17 signaling pathway (Fig. 5B, C).

**Figure 6.**
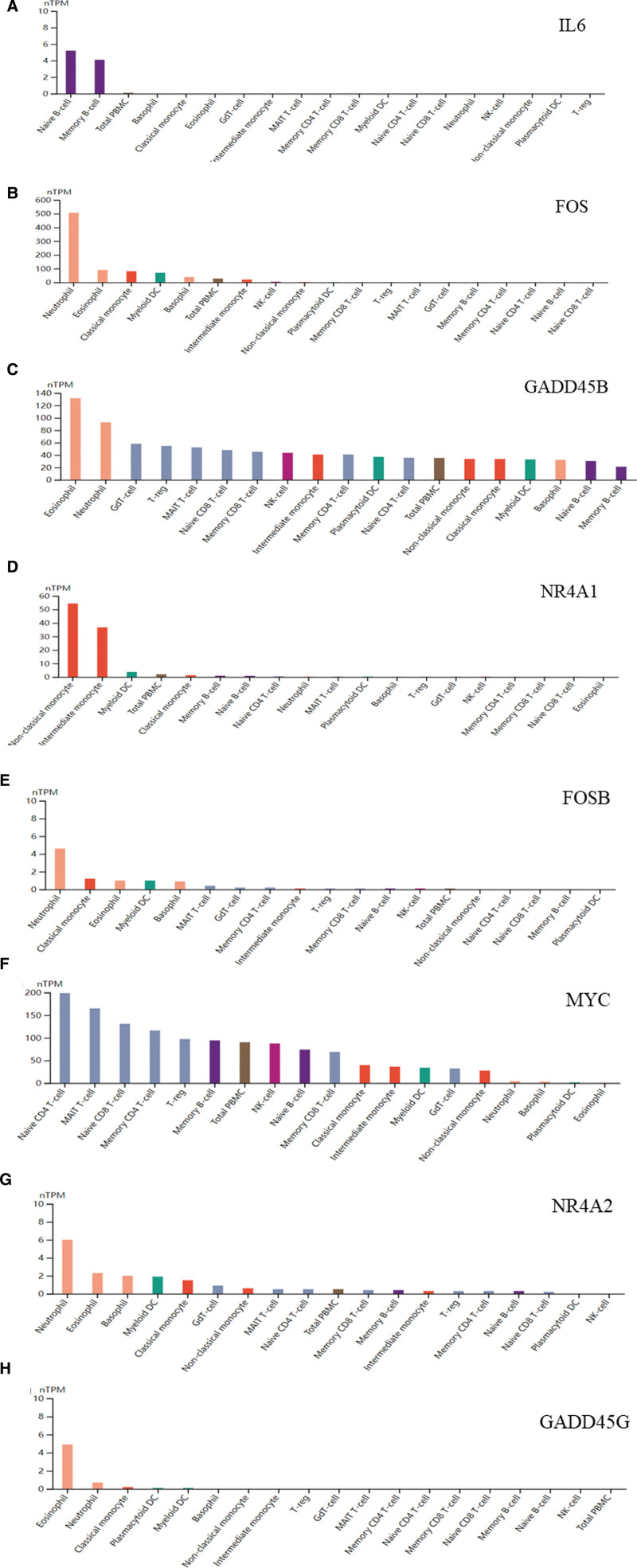
Expression of 8 hub genes in immune cells. FOS = Fos Proto-Oncogene, AP-1 Transcription Factor Subunit, FOSB = FosB Proto-Oncogene, AP-1 Transcription Factor Subunit, GADD45B = Growth Arrest and DNA Damage Inducible Beta, GADD45G = Growth Arrest and DNA Damage Inducible Gamma, GO = Gene ontology, IL = interleukin, KEGG = Kyoto Encyclopedia of Genes and Genomes, MYC = MYC Proto-Oncogene, NR4A1= Nuclear Receptor Subfamily 4 Group A Member 1, NR4A2 = Nuclear Receptor Subfamily 4 Group A Member 2, PPI = protein–protein interaction network.

**Figure 7.**
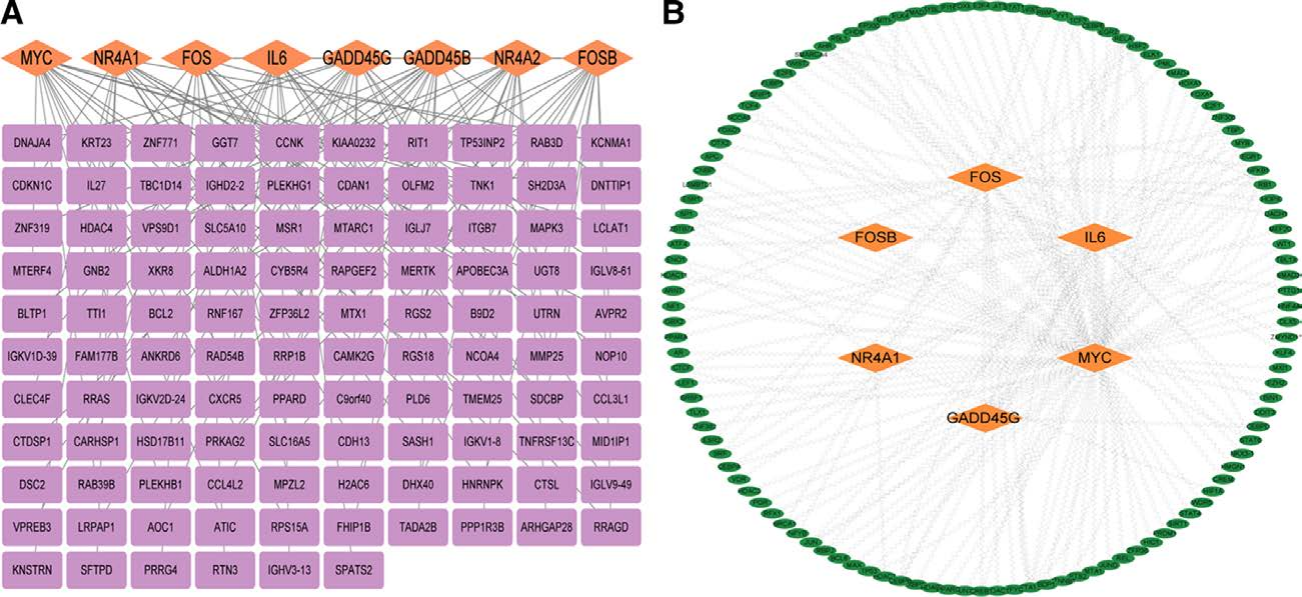
Prediction of adjacent genes and transcription factors associated with hub genes. (A) Prediction of adjacent genes associated with hub genes. Purple rectangles represent adjacent genes related to hub genes, and orange-yellow diamonds represent hub genes. (B) Transcription factor prediction of regulatory hub genes. Green ovals represent transcription factors, while orange-yellow diamonds represent hub genes. FOS = Fos Proto-Oncogene, AP-1 Transcription Factor Subunit, FOSB = FosB Proto-Oncogene, AP-1 Transcription Factor Subunit, GADD45B = Growth Arrest and DNA Damage Inducible Beta, GADD45G = Growth Arrest and DNA Damage Inducible Gamma, GO = Gene ontology, IL6 = interleukin 6, KEGG = Kyoto Encyclopedia of Genes and Genomes, MYC = MYC Proto-Oncogene, NR4A1= Nuclear Receptor Subfamily 4 Group A Member 1, NR4A2 = Nuclear Receptor Subfamily 4 Group A Member 2, PPI = protein–protein interaction network.

Subsequently, the expression of 8 hub genes in various immune cells was searched in the HPA database, and it was found that neutropil and eosinophil were the cells with the highest expression of hub genes (Fig. 6A–H). The genes that interacted with 8 hub genes were searched in the HPA database, and the hub gene-interacted target network was constructed by CytoScape 3.7.0 (Fig. 7A).

### 3.5. TF prediction of hub genes

The TTRUST database contains 120 TFs that regulate the 6 key genes, of which STAT3, NFKB1, RELA, AHR, RB1, and SP1 were the major ones (Fig. 7B, Table 1).

**Table 1:** Topological parameters of the network of transcription factors and hub genes.

Name	Betweenness centrality	Closeness centrality	Neighborhood connectivity	Radiality	Topological coefficient	Degree
STAT3	0.009707228	0.480468750	45.66666667	0.985582656	0.397101449	7
NFKB1	0.014669688	0.447272727	36.33333333	0.983523035	0.378947368	7
RELA	0.014669688	0.447272727	36.33333333	0.983523035	0.378947368	7
AHR	0.009707228	0.480468750	45.66666667	0.985582656	0.397101449	4
RB1	0	0.447272727	53.50000000	0.983523035	0.551546392	4
SP1	0.088342441	0.500000000	35.50000000	0.986666667	0.296218487	4

### 3.6. Prediction of drug candidates from hub genes

**Figure 8.**
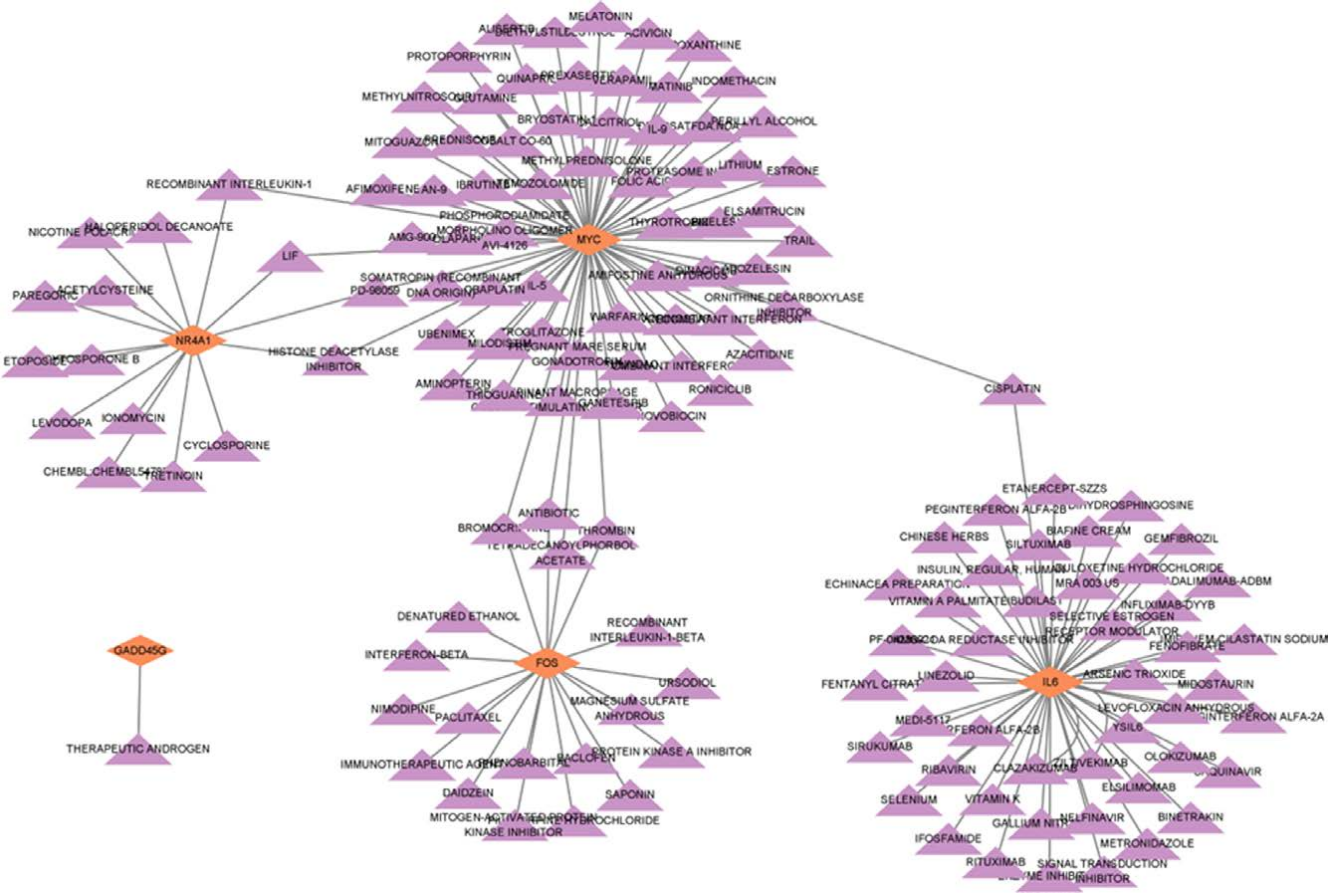
Prediction of drug candidates for hub genes. Purple triangles represent drugs that bind tightly to hub genes, and orange diamonds represent hub genes. FOS = Fos Proto-Oncogene, GADD45G = Growth Arrest and DNA Damage Inducible Gamma, IL6 = interleukin 6, MYC = MYC Proto-Oncogene, NR4A1= Nuclear Receptor Subfamily 4 Group A Member 1.

Eight hub genes were further studied and screened for drugs for the treatment of NASH caused by T2DM. The retrieved data were visualized and analyzed (Fig. 8). One hundred forty-seven targets and 153 edges were involved in the network. To visually reflect the key candidates, the top 15 drugs with the highest interaction scores were selected for presentation (Table 2). Thyrotropin was identified by NR4A1. Nimodipine and bromocriptine were identified by MYC. Siltuximab, magnesium sulfate anhydrous, vitamin A palmitate, baclofen, metronidazole, and echinacea preparation were identified by FOS. Quinapril, acetylcysteine, pilocarpine hydrochloride, ursodiol, nelfinavir, and levofloxacin anhydrous were identified by IL6.

**Table 2:** Prediction of drug candidates.

No.	Gene	Drug	Regulatory approval	Interaction score
1	IL6	Quinapril	Approved	2.407349
2	FOS	Siltuximab	Approved	1.552107
3	IL6	Acetylcysteine	Approved	0.80245
4	FOS	Magnesium sulfate anhydrous	Approved	0.620843
5	IL6	Pilocarpine hydrochloride	Approved	0.601837
6	IL6	Ursodiol	Approved	0.601837
7	MYC	Nimodipine	Approved	0.569856
8	NR4A1	Thyrotropin	Approved	0.524267
9	FOS	Vitamin A palmitate	Approved	0.443459
10	FOS	Baclofen	Approved	0.413895
11	IL6	Nelfinavir	Approved	0.401225
12	IL6	Levofloxacin anhydrous	Approved	0.401225
13	FOS	Metronidazole	Approved	0.388027
14	FOS	Echinacea preparation	Approved	0.388027
15	MYC	Bromocriptine	Approved	0.366336

FOS = Fos Proto-Oncogene, IL6 = interleukin 6, MYC = MYC Proto-Oncogene, NR4A1= Nuclear Receptor Subfamily 4 Group A Member 1.

## 4. Discussion

NAFLD has gradually become the most common chronic liver disease in the world. A large number of studies have confirmed that T2DM is one of the most important risk factors for NAFLD and accelerates the progression of NAFLD. The incidence of NASH in T2DM patients worldwide is 37.3% (95% confidence level: 24.7%–50.0%), which has attracted the attention of many researchers.^[23,24]^ However, the correlation between T2DM and NASH is still unclear.

In this study, we used bioinformatics methods to mine 55 DEGs from the 2 datasets, including 32 upregulated genes and 23 downregulated genes, and they overlapped with 17,521 T2DM-related targets, and 46 common targets were obtained. Subsequently, GO and KEGG enrichment analyses were performed on 46 common targets, which were mainly involved in the response to GCs and tumor necrosis factor and in the regulation of multicellular growth, oxidoreductase-reactive oxygen species uptake, T cell proliferation, apoptosis, amine transport, lipid, and other biological processes. signaling pathways were mainly enriched in colorectal cancer, amphetamine addition, the PPAR signaling pathway, and the toll-like receptor signaling pathway. More and more epidemiological and experimental evidence has shown that GCs are closely related to NASH. In the fasting state, GCs can mobilize lipids and increase the delivery of fatty acids. In the eating state, GCs can promote lipid accumulation and increase lipid accumulation in the liver, thereby causing benign hepatic steatosis to become steatohepatitis.^[25,26]^ The occurrence of NASH is regulated by many pro-inflammatory factors, among which tumor necrosis factor-α is involved in the regulation of hepatocyte inflammation, cell death, and fibrosis and plays an important role in the progression of NASH.^[27]^ One study reported that oxidative stress can promote the progression of NASH and induce the occurrence of LF.^[28]^ Studies have found that NASH is characterized by immune cell infiltration, and T cells are highly expressed in the mouse NASH model.^[29]^ PPAR plays an important role in maintaining lipid and glucose homeostasis and is also involved in the regulation of the inflammatory response. There is evidence that PPAR can promote the occurrence and progress of NASH.^[30,31]^ Toll-like receptors are involved in the regulation of a series of inflammatory responses, and the most typical features of NASH are lipid accumulation and inflammatory cell infiltration in hepatocytes. Li et al^[32]^ found that by inhibiting the toll-like receptor-4/nuclear transcription factor-κB p65 signaling pathway, the inflammatory response and apoptosis of hepatocytes can be reduced.

Eight hub genes were identified by PPI network analysis, namely IL6, FOS, GADD45B, NR4A1, FOSB, MYC, NR4A2, and GADD45G. Ali et al^[33]^ reported that ILs play a crucial role in the pathogenesis of liver diseases, and ILs are involved in the progression and control of liver diseases by regulating cell signaling pathways. Bocsan et al^[34]^ used enzyme linked immunosorbent assay to measure the plasma IL6 level in 66 NASH patients and 30 healthy volunteers and found that the IL6 level in NASH patients was significantly increased, suggesting that IL6 is involved in the regulation of the development process of NASH. FOS and FOSB belong to the activator protein 1 family and are involved in cell proliferation, migration, transformation, invasion, and death.^[35]^ Yu et al^[36]^ studied the role of activator protein 1 in the regulation of kidney and liver inflammation in mice and found that the gene knockdown of FOS, a family member, significantly reduced the inflammatory response in the kidney and liver. Cai et al^[37]^ studied the regulatory mechanism of microRNA-29 in NASH and found that microRNA-29 affected NASH through the IL-17 signaling pathway after acting on FOS. The results of quantitative real-time polymerase chain reaction and Western blot data analysis showed that FOS was highly expressed in NASH mice and showed the same trend.^[37]^ GADD45B and GADD45G are members of the growth arrest DNA damage-inducible gene (GADD45) family, respectively, which are involved in cell cycle arrest, DNA repair, apoptosis, innate immunity, and genome stability. Studies have found that GADD45B gene deficiency can accelerate cell senescence and reduce LF.^[38]^ Dong et al^[39]^ found that overexpression of GADD45β by AAV8-mediated gene transfer in a high-fat high-fructose diet-fed mouse model reduced serum and hepatic triglyceride levels and alleviated insulin resistance. NR4A1 and NR4A2 are members of the NR4A family of nuclear receptors, which are a group of early genes induced by peptide hormones, growth factors, cytokines, and inflammatory stimuli. As effective sensors of changes in the cellular microenvironment, NR4A1 and NR4A2 control metabolism, cardiovascular, and neurological functions and also take into account the homeostasis of immune cells in inflammation and cancer.^[40]^ Sun et al^[41]^ used hyperoside in mice fed with a high-fat diet (HFD) and found that hyperoside regulated macrophage polarization through NR4A1, thereby having a protective effect on HFD-induced NAFLD mice, with significant reductions in steatosis, insulin resistance, and inflammatory response. When Li et al^[42]^ studied the progression of steatosis and the molecular mechanism of NASH, they found that miR-145a-5p could down-regulate NR4A2, inhibit the expression of NASH-related genes, and reduce the progression of liver inflammation, liver injury, and fibrosis. Similarly, NR4A2 overexpression was found to promote the progression of steatosis to NASH, while specific knockdown of NR4A2 in the liver protected mice from diet-induced NASH. The MYC gene, which consists of 3 paralogenics of C-MYC, N-MYC, and L-MYC, is a TF with a wide range of biological functions, particularly in the control of cell proliferation.^[43]^ However, many studies focus on tumor growth and reproduction, and there are few studies on other diseases. One study reported that intestinal MYC expression followed the same trend as body mass index increase, and specific knockdown of intestinal MYC in mice ameliorated HFD-induced obesity, insulin resistance, and hepatic steatosis and steatohepatitis.^[44]^ The above 8 hub genes are closely related to the process of NASH, which confirms that our results are consistent with those reported in the literature.

From the hub gene enrichment results, T2DM affected the progression of NASH possibly by regulating DNA transcription activator activity, the apoptosis process, and in response to GCs and exogenous stimuli. The GC stress hormone drives fat metabolism in the liver, but complete blocking and stimulation of the GC signal will aggravate NAFLD pathology, while NASH is usually caused by abnormal fat metabolism in the liver, continuous accumulation, and degeneration, leading to a series of inflammatory reactions. Therefore, the development process of NASH is closely related to GC. KEGG enrichment analysis showed that the hub genes were concentrated in colon cancer and the IL-17 signaling pathway, and colon cancer may be associated with NASH. Many studies^[45–48]^ have confirmed that the IL-17 signaling pathway mediates NASH progression. In order to clarify the correlation between the extracted hub gene and immune response, we searched the hub gene in the HPA database and found that its expression in neutrophils and eosinophils was higher than that of other immune cell drugs. Neutrophils are usually the first responders to acute inflammation and form a variety of barrier mechanisms through phagocytosis, cytokine secretion, reactive oxygen species production, and neutrophilic traps, which help improve the body’s resistance to external toxic substances.^[49]^ Van der Windt et al^[50]^ found that inhibiting the formation of neutrophilic traps in mice would not affect the development of fatty liver but would change the process of NASH and eventually inhibit the growth of liver tumors. Eosinophils are components of white blood cells, which have the function of killing bacteria and parasites and are also extremely important cells in the process of immune response and allergic reaction. Eosinophils can release the contents of the particles, causing tissue damage and promoting the progression of inflammation.^[51]^ Adipose tissue eosinophils regulate adipose tissue homeostasis and systemic low-grade inflammation through IL4.^[52]^ In addition, our study also predicted the TFs of hub genes, and these TFs allow us to further explore the regulation of the development of NASH by T2DM.

In this study, 15 candidate drugs were identified by hub genes, including quinapril, siltuximab, acetylcysteine, magnesium sulfate anhydrous, pilocarpine hydrochloride, ursodiol, nimodipine, thyrotropin, vitamin A palmitate, baclofen, nelfinavir, levofloxacin anhydrous, metronidazole, echinacea preparation, and bromocriptine. Among these 15 drugs, levofloxacin anhydrous and metronidazole belong to the antibacterial agents and should be excluded. Thyrotropin and bromocriptine are hormonal modifiers and should be excluded. Quinapril is a new second-generation angiotensin-converting enzyme inhibitor that is mainly used for chronic heart failure, hypertension, and nephrotic syndrome. It has little correlation with NASH, so it should be excluded.^[53]^ Similarly, magnesium sulfate anhydrous, nimodipine, vitamin A palmitate, baclofen, and nelfinavir have not been reported to be associated with NASH treatment and should be excluded. Contrary to the above, siltuximab,^[54]^ acetylcysteine,^[55]^ pilocarpine hydrochloride,^[56]^ ursodiol,^[57]^ and echinacea^[58]^ have been proposed as potential drugs for NASH in previous studies. Even so, the efficacy of these 5 drug candidates in the treatment of T2DM-induced NASH needs to be further studied.

To sum up, our study showed that the effects of T2DM on NASH progression were multifactorial, including changes in hormone levels, immune response, regulation of cell proliferation, and oxidation reduction reactions. Unfortunately, our study still has some shortcomings: The sample size in the public database is too small, and a supplementary sample size would be more comprehensive. The predicted results have not been verified, and further clinical reports or experimental studies are needed to support our conclusions.

## 5. Conclusion

IL6, FOS, GADD45B, NR4A1, FOSB, MYC, NR4A2, and GADD45G are the 8 hub genes for T2DM and NASH, which provide new approaches and potential targets for the treatment of T2DM-induced NASH in the future. In addition, we predict candidate drugs associated with NASH that are promising as potential agents for the co-occurrence of the 2 diseases. In the future, we will further study the correlation between T2DM, GCs, and NASH so as to obtain a more complete pharmacological mechanism.

## Acknowledgments

We thank Xuemei Li for contributing to the data processing of this study, although she is not listed in the author's information.

## Author contributions

**Methodology:** Bo Wu, Yuekun Wang.

**Software:** Bo Wu, Xiaohong Lan, Wei Wei, Yuekun Wang, Yang Yang, Zhiyang Yu, Min Huang.

**Writing – original draft:** Bo Wu.

**Writing – review & editing:** Bo Wu, Qinyan Wu.

**Supervision:** Xiaohong Lan.

**Data curation:** Ming Gao.

**Visualization:** Wei Wei, Qinyan Wu.

**Resources:** Zhiyang Yu.

**Validation:** Min Huang.
